# Knowledge and Attitude Regarding Patients Right among Nurses in a Teaching Hospital: A Descriptive Cross-sectional Study

**DOI:** 10.31729/jnma.4661

**Published:** 2020-02-29

**Authors:** Tara Gurung, Srijana Ghimire

**Affiliations:** 1 Department of Medicine, Chitwan Medical College and Teaching Hospital, Bharatpur, Chitwan, Nepal; 2 School of Nursing, Chitwan Medical College and Teaching Hospital, Kailashnagar, Chitwan, Neapl

**Keywords:** *attitude*, *knowledge*, *patients right*

## Abstract

**Introduction:**

It is important to maintain trust and satisfaction among patients. The health personnel take an important role to overcome their right. The objective of this study was to find out knowledge and attitude regarding patients' rights among nurses in Teaching Hospital.

**Methods:**

A descriptive cross sectional study was conducted among 122 nurses in different wards of Teaching Hospital. Nurses were selected by using simple random sampling technique for data collection. Ethical clearance was taken from Chitwan Medical College institutional reviewers Committee (CMC-IRC) to conduct the study. A structured, self- administered questionnaire and five-point Likert scale were used to analyze the collected data. Data was collected from 27^th^ Ashadh to 9^th^ Shrawan 2075.

**Results:**

This study revealed that out of 122 respondents, 30 (24.6%) of respondents have an adequate level of knowledge whereas about half 62 (50.8%) of respondents had favorable and 60 (49.2%) had an unfavorable level of attitude regarding patients right. Sixty-one (50%) of the nurses were from the age group <22 years, 27 (77.9%) were unmarried, about 93 (76.2%) of nurses had completed Proficiency Certificate Level Nursing, 101 (82.2%) had work experience less than 24 months.

**Conclusions:**

According to the study, it concluded that one-fourth of the respondents have an adequate level of knowledge, one-half of the respondents had a favorable attitude. Therefore, knowledge and attitude regarding patients' rights should be increase through in-service education and seminars should be organized by the administration to promote quality health care service.

## INTRODUCTION

The patient right is defined as the rule of conduct between people who beneft from health services and institution who provide them and is owed by the patient by physician and state. Condition and protection of this right related to nursing profession.^1^

Health care personals as doctors, health assistance and most especially nurses are always in close contact with the patient as nurses remain with the patient all the time to provide care. For the quality of care, provision nurses should have not only the knowledge of disease but also should have the knowledge and be aware of human rights, abuses to the patient that may occur in the hospital while providing care. So nurses must play an active role in eradicating such usual or unusual abuses and preventing them from occurring within health institutions. And here this will require a nurse to be knowledgeable an awareness regarding patient's right.^2^

The study was done among nurses of Chitwan Medical College Teaching Hospital in order to find out the knowledge and attitude regarding patients' rights.

## METHODS

The descriptive cross-sectional research design was done at the ICU, orthopedic ward, OT, pediatric, respiratory, medicine, ENT, surgery, emergency and gyne/obstetric department of Chitwan Medical College Teaching Hospital (CMCTH), from 27^th^ Ashadh to 9^th^ Shrawan 2075. Ethical clearance was taken from Chitwan Medical College institutional reviewers Committee (CMC-IRC) to conduct the study. All the nurses who were working in the post of staff nurse, senior staff nurse, and nursing officers were included in the population of the study and nursing supervisor, matron was excluded because they mostly involve in administrative activities. The nurse of CCU and Dialysis was excluded because pretesting was done in that area. Staff nurse, senior staff nurse, and nursing officers were included because most of all they involve inpatient care, ward activities. Written informed consent was obtained from each respondent by clarifying the purpose of the study prior to data collection. Simple random sampling technique was used through the name list of nurses to select the desired sample.

Sample size estimation:

n_0_= Z^2^ × p × q/ e^2^

   = 1.96^2^ × 0.68 × 0.32/ 0.7^2^

   = 170

where,

p = prevalence of previous study^2^ (Thapa and Samson)

q= 1-p,

e = margin of error, 7%

Z= 1.96 at 95 % CI

For definite population,

n = n_0_/ 1+ (n_0_-1)/N = 170/1+ 170-1

            342

   = 114

Adding, 5% non-response rate, sample was calculated to be 120.

where,

N= Total population i.e. 342 nurses

Therefore, the total sample size (n) was 122.

Structured self-administered questionnaire was used to identify the level of knowledge of nurses developed based on research of^3-4^ and 5 points Likert scale was used to identify level of attitude regarding patients' right among nurses developed based on research study of^5-8^ Aka and her team as well as via literature review.

All collected data were reviewed and checked for completeness, consistency and accuracy. Coded data were entered in EPI data 3.1. The entered data was exported into IBM SPSS version 20. Data were analyzed by using descriptive statistics (frequency, percentage, mean and standard deviation). The findings of the study were presented in tables.

The research instrument consisted of three parts:

Part I- Question-related to socio-demographic data

Part II- Question-related to knowledge regarding patients right among nurses.

Part III- Question-related to attitude regarding patients' rights among nurses.

## RESULTS

Out of 122 respondents, 61 (50%) of respondents belong to group of <22 years, 77.9% respondents were unmarried, 91% of respondents live in gaupalika, 84 (68.9%) belongs to ethnic group brahmin where as 13.9% belongs to dalit. In regard to source of information about 96 (78.7%) of respondent get information from book regarding the patient right (Table 1).

**Table 1:** Socio-demographic characteristic of the respondents.

Variables	n (%)
Age ( In completed years)	
<22yrs	
≥22yrs	61 (50.0)
(Mean(SD)= 22.09(2.412), Min=18, Max=30)	61 (50.0)
Marital status of respondents	
Married	27 (22.1)
Unmarried	95 (77.9)
Place of residents of respondents	
Gaupalika	11 (9.0)
Municipality	11 (91.0)
Ethnicity of respondents	
Brahmin	84 (68.9)
Janajati	22 (18.0)
Newar	11 (9.0)
Muslim	3 (2.5)
Dalit	2 (1.6)
Sources of information	
Books	96 (78.7)
Friends	29 (23.8)
Curriculum	31 (25.4)
Internet	29 (23.8)
Seniors	17 (13.9)

Professional and organizational characteristics of respondents in which greater than two third 93 (76.2%) of respondent had completed Pcl Nursing and majority 114 (93.4%) of respondents were working as staff nurse. Regarding the work experience, majority (82.8%) has less than 24 months whereas 77% of respondent working in current ward for less than 18 months. More than half (58.2%) of the respondents have not receive in-service education regarding client right and 82% of the respondents have habit of self directed learning. about half (53.3%) of respondents face problem regarding patients right (Table 2).

**Table 2:** Professional characteristics of the respondents.

Variable	n (%)
Professional qualification	
PCL Nursing	93 (76.2)
B.Sc.Nursing	19 (15.6)
BNS	10 (8.2)
Professional designation	
Staff Nurse	114 (93.5)
Senior Staff Nurse	7 (5.7)
Nursing Officer	1 (0.8)
Hospital Experience	
<3 Month	18 (14.8)
3-24 Month	83 (68.0)
>24 Month	21 (17.2)
(Mean =15.71,Min=1,Max=120)	
Current Ward Experience	
<3 Month	
3-18 Month	23 (18.8)
>18 Month	71 (58.2)
(Mean =11.71,Min=1,Max=120)	28 (23.0)
In-service Education regarding patient right	
Yes	51 (41.8)
No	71 (58.2)
Self directed learning regarding patient right	
Yes	100 (82.0)
No	22 (18.0)
Face any problem regarding patient right	
Yes	65 (53.3)
No	57 (46.7)

The level of knowledge regarding patient right, 24.6% had adequate knowledge (Table 5). Out of total score 14, the maximum score obtained by the respondent was 14 and minimum score was 2 (Table 3).

**Table 3:** Level of knowledge regarding patient's right of the respondents.

Level of knowledge	n (%)
Adequate (>8)	30 (24.6)
Inadequate(≤ 8)	92 (75.4)

Sixty two (52.8%) of the respondents had satisfactory attitude regarding patients right (Table 4).

**Table 4:** Level of attitude regarding patient's right of the respondents.

Level of attitude	n (%)
Favorable (≥62)	62 (50.8)
Unfavorable (<62)	60 (49.2)

**Table 5:** Level of knowledge of patient's right and selected variables.

Variables	Level of knowledge Adequate n (%)	Inadequate n (%)
Age (In Years)		
<22	19 (31.1)	42 (68.9)
≥22	11 (18.0)	50 (82)
Marital status		
Married	8 (29.6)	19 (70.4)
Unmarried	22 (23.2)	73 (76.8)
Place of residence	
Gaupalika	4 (36.4)	7 (63.6)
Municipality	26 (23.4)	85 (76.6)
Ethnic Group	
Brahmin/Chhetri	21 (25.0)	63 (75.0)
Others	9 (23.7)	29 (76.3)
Professional qualifcation	
PCL	26 (28.0)	67 (72.0)
BSc.Nsg & BNS	4 (13.8)	25 (86.2)
Professional Designation	
Staff Nurse	70 (61.4)	44 (38.6)
Senior Staff Nurse& NO	3 (37.5)	25 (62.5)
Month of Experience in Hospital	
<12 Month	16 (26.2)	45 (73.8)
≥12 Month	14 (23.0)	47 (77.0)
Current Ward Experience on time of data collection	
<8 Month ≥8Month	11 (18.6) 19 (30.2)	48 (81.4) 44 (69.8)
In-service Education about patient right	
Yes	14 (27.5)	37 (72.5)
No	16 (22.5)	55 (77.5)
Self-Directed Learning	
Yes	26 (26.0)	74 (74.0)
No	4 (18.2)	18 (81.8)

**Table 6:** Level of Attitude of Patient Right and Selected Variables.

Variables	Level of Attitude Satisfactory n (%)	Unsatisfactory n (%)
Age ( In Years)		
<22	30 (49.2)	31 (50.8)
≥22	32 (52.5)	29 (47.5)
Marital status		
Married	13 (48.15)	14 (51.9)
Unmarried	49 (51.6)	46 (48.4)
Place of residence		
Gaupalika	7 (63.6)	4 (36.4)
Municipality	55 (49.5)	56 (50.5)
Ethnic group		
Brahmin/ Chhetri	40 (47.6)	44 (52.4)
Others	22 (57.9)	16 (42.1)
Professional qualification		
PCL	47 (50.5)	46 (49.5)
B.Sc.Nsg. & BNS	15 (51.7)	14 (48.3)
Professional designation		
Staff Nurse	59 (51.8))	55 (48.2)
Senior SN & NO	3 (37.5)	5 (62.5)
Month of experience in hospital		
<18 Month	31 (50.8)	30 (49.2)
≥18 Month	31 (50.8)	30 (49.2)
Current ward experience on time of data collection
<8 Month	32 (54.2)	27 (45.8)
≥8 Month	30 (47.6)	33 (53.4)
Self-directed learning	
Yes	49 (49)	51 (51)
No	13 (59.1)	9 (40.9)

## DISCUSSION

The study population consists of 122 nurses working in all wards of Chitwan Medical College Teaching Hospital, Bharatpur.

This study revealed that out of 122 respondents, 30 (24.6%) of respondent have adequate level of knowledge regarding patients' right and 92 (75.4%) of respondents have inadequate level of knowledge which is not the good result for nursing like profession as their low knowledge effect on their practice which consequently effects on patient wellbeing. Poor knowledge may be due to not utilizing the knowledge in practice when dealing with the patient. There is no statistically significant relationship between knowledge with age, marital status, place of residence, ethnic group, professional qualification, professional designation, experience in hospital, experience in the current ward, in-service education and self-directed learning. Majority 101 (80%) of the respondents have working experience of fewer than 24 months, which may be the cause that no association of working experience may occur. This study is a contrast to the study conducted by Nejad et al, who revealed that there is an association between knowledge of the nurse with their working experience and working hospital either private or governmental.^3^

Regarding patients' right about half 62 (50.8%) of respondents had a favorable level of attitude and 60 (49.2%) had an unfavorable level of attitude it may be due to poor knowledge and more workload of nursing staff. Similarly, a study conducted in Turkey shows that attitude towards patient rights differs for every patient ranging from 35.8 to 98.1%.

## CONCLUSIONS

The findings of the study concluded that one-fourth of the respondents have an adequate level of knowledge, one-half of the respondents has a favorable attitude.

## Conflicts of Interest:

None.

## References

[1] Sardar T, Qasim AP, Majeed I, Afzal M, Waqas A, Gillani SA (2017). Knowledge, attitude and practices of the nurses regarding patient rights: a study in Punjab Institute of Cardiology, Lahore. Ann Punjab Med Coll..

[2] Thapa K, Samson VW (2017). A study to assess the knowledge and attitude of staff nurses regarding human rights of mentally ill patients at selected hospitals of Bangalore, India. Journal of Kathmandu Medical College..

[3] Mohammed Nejad E, Begjani J, Abotalebi G, Salari A, Ehsani SR (2011). Nurses awareness of patients rights in a teaching hospital.. J Med Ethics Hist Med..

[4] Akca SO, Akpinar YY, Habbani T (2015). Knowledge and attitudes of nurses regarding patient rights: a Corum/Turkey sample. Rev Assoc Med Bras (1992).

[5] Al-Bardah SH, Shenouda MS, Magdy H (2012). Assessment of nurses' awareness regarding mother and child rights at Al-Sabaeen Hospital in Sana’a-Yemen.. Med J Cairo Univ..

[6] El-Sobkey SB, Almoajel AM, Al-Muammar MN (2014). Knowledge and attitude of Saudi health professions’ students regarding patient’s bill of rights. Int J Health Policy Manag..

[7] Khalaf SK, Al-Asadi JN, Abed Ah, Shami SA, Al-Shamry H (2014). Knowledge and attitudes towards patient’s rights among health care providers in primary care health centers in Basrah.. Int J Med Res Pharm Sci..

[8] Utkualp N, Yildiz H (2016). Awareness and attitudes of nurses working in a university hospital on patients' rights. International Journal of Caring Sciences..

